# Bionic Inner-Tapered Energy Absorption Tube Featuring Progressively Enhanced Fold Deformation Mode

**DOI:** 10.3390/biomimetics10010006

**Published:** 2024-12-26

**Authors:** Shuang Zhang, Zhengzhi Mu, Wenda Song, Zhiyan Zhang, Hexuan Yu, Binjie Zhang, Zhiwu Han, Luquan Ren

**Affiliations:** 1 Key Laboratory of Bionic Engineering, Ministry of Education, Jilin University, Changchun 130022, China; zhangshuang22@mails.jlu.edu.cn (S.Z.); songwd@jlu.edu.cn (W.S.); zhiyan22@mails.jlu.edu.cn (Z.Z.); yuhx22@mails.jlu.edu.cn (H.Y.);; 2 Institute of Structured and Architected Materials, Liaoning Academy of Materials, Shenyang 110167, China; 3 Nanjing Drum Tower Hospital, Affiliated Hospital of Medical School, Nanjing University, Nanjing 210093, China; zhangbj1995@163.com

**Keywords:** energy absorption tube, inner-tapered, bionic design, bamboo internode

## Abstract

Slender tubes are in high demand owing to their lightweight and outstanding energy absorption. However, conventional slender tubes are prone to catastrophic failures such as Euler’s buckling under axial load. Interestingly, growing bamboos overcome this similar dilemma via a unique tapered intine in the internodes, which endows them with excellent energy absorption. Inspired by this finding, a bionic inner-tapered tube (BITT) was designed to enhance the energy absorption of slender tubes under axial load. The special energy absorption (SEA) was evaluated via a quasi-static axial compression test. Then, theoretical calculation and finite element analysis were carried out to analyze the energy absorption mechanisms. The results reveal that the tapered inner wall induces a progressively enhanced fold deformation mode for BITT, which not only prevents buckling failure and decreases initial peak crushing load but also improves the energy absorption efficiency by increasing plastic deformation. The influences of taper and length–diameter ratio on the axial energy absorption of BITT are explored. Finally, the bionic square array (BSA) and bionic hexagon array (BHA) are fabricated by taking BITT as the basic structural unit, which significantly improves the main energy absorption performance indicators under axial load.

## 1. Introduction

Slender tubes are widely used in key engineering fields such as the undercarriages of aircraft, the landing legs of rocket landers, and the girders of architecture due to their lightweight and outstanding energy absorption. The length of the slender tube is much larger than the other dimensions, so a non-negligible defect arises, the structure is unstable under axial load and prone to catastrophic failures such as Euler’s buckling [1,2]. Even if the stress of the structure is far below the yield strength of the substrate material, catastrophic failure will also occur with a high probability [3,4,5]. This phenomenon has seriously puzzled engineers in related fields and caused a severe economic loss.

In nature, there are also many natural tubular structures with a large slenderness ratio. After their evolution for thousands of years, they can grow healthily even under harsh load and exhibit completely different mechanical behavior from conventional engineering tubes [6,7]. Recently, many bionic slender tubes have been designed for energy absorption, which mainly include multi-cell tube [8,9,10], foam-filled tube [11,12], and other tubes with complex cross sections [13,14]. The axial energy absorption of these slender structures has been improved to a more excellent extent. However, due to the complexity of the bionic structure, the actual sample is unable to fully achieve the expected effect due to the precision limitation of the fabrication process, which makes it difficult to be widely used as an engineering structure. As the most typical natural hollow tubular structure, bamboo can withstand gravity, wind, rain, and the impact load from fierce animals as well as maintain upright growth with a large slenderness ratio. It has excellent toughness which is comparable to alloy materials in spite of its multi-nodal structure [15,16]. In the 1990s, Amada et al. first suggested that bamboo could be regarded as a composite material reinforced axially by bundle sheath and had hierarchical gradient structures at the macroscopic and microscopic scale [17]. Then, more researchers have explored the superior performance of bamboo. Palombini et al. applied X-ray microtomography to characterize the 3D microstructure of bamboo and used numerical analysis to investigate its mechanical properties. The results demonstrated bamboo’s superior mechanical performance, particularly in compression and bending, supporting its future use in engineering design [18]. Tan et al. combined finite element analysis and experimental testing to investigate the mechanical properties of functionally graded hierarchical bamboo structures. This study demonstrates that the functionally graded structure significantly improves the mechanical properties of bamboo, especially in terms of compressive and bending strength [19]. Hongbo et al. investigated the mechanical properties of bamboo and its vascular bundles through material testing and microscopic structural analysis. The results show that the vascular bundles of bamboo play a significant role in enhancing its mechanical properties, improving both tensile and compressive strength [20]. Taylor et al. combined experimental mechanics methods and finite element simulations to analyze the biomechanics of bamboo nodes. Their study demonstrates that bamboo nodes play a crucial role in the overall mechanical performance, with particularly strong contributions to bending strength [21]. Si-Ming et al. used mechanics testing and microstructural analysis to investigate the mechanical properties of bamboo nodes and design nodes with a hierarchical fibrous structure. The study demonstrates that the hierarchical fibrous structure significantly enhances the mechanical strength of bamboo nodes, especially in compressive and bending performance [22]. These studies provide a comprehensive analysis of the multi-scale structural characteristics of bamboo and investigate the mechanisms by which these structural features contribute to its outstanding mechanical properties.

Based on the function–structure interaction mechanism of bamboo, many cutting-edge designs have been developed to improve the energy absorption of impact-resistant structures. Ma et al. studied the elastic buckling behavior of bionic cylindrical shells based on bamboo through theoretical analysis and numerical simulations. Their results show that the unique structure of bamboo effectively enhances the elastic buckling performance of cylindrical shells [23]. Fu et al. investigated the energy absorption performance of bionic bamboo thin-walled structures using finite element simulations and experimental studies. This study demonstrates that the bionic bamboo thin-walled structures exhibit excellent energy absorption capabilities, making them suitable for collision and impact applications [24]. Chen et al. conducted experimental studies to investigate the energy absorption performance of bionic bamboo tubes under axial crushing. Their results show that bionic bamboo tubes exhibit excellent energy absorption capabilities under axial compression, making them suitable for protective structure designs [25]. Liang et al. investigated the design and performance of bionic bamboo tubes under multiple compression load cases using numerical simulations and experimental methods. Their study proposes a novel bionic bamboo tube design that demonstrates excellent energy absorption performance under various compression load conditions [26]. Hu et al. designed a bionic honeycomb tubular-nested structure inspired by bamboo and investigated their energy absorption characteristics under axial crushing. The results demonstrate that the bionic bamboo-inspired honeycomb tubular-nested structure exhibits excellent energy absorption characteristics [27]. Through the analysis of the above studies, bionic structures inspired by the various structural characteristics of bamboo exhibit excellent energy absorption properties under different types of loads, especially under axial impact. Meanwhile, the combination of finite element analysis and experiments has been proven to be an effective method for studying the performance of bionic impact-resistant structures. It is found that the above studies mainly explored the vital role of bamboo microstructures on the excellent mechanical properties of bamboos. However, for every bamboo stalk with large slenderness ratio, the mechanical properties of internodes as the basic macrostructure unit directly determine the overall performance of a bamboo. The influence of internode macrostructural characteristics on the mechanical properties of the bamboo is equally non-negligible.

**Figure 1:**
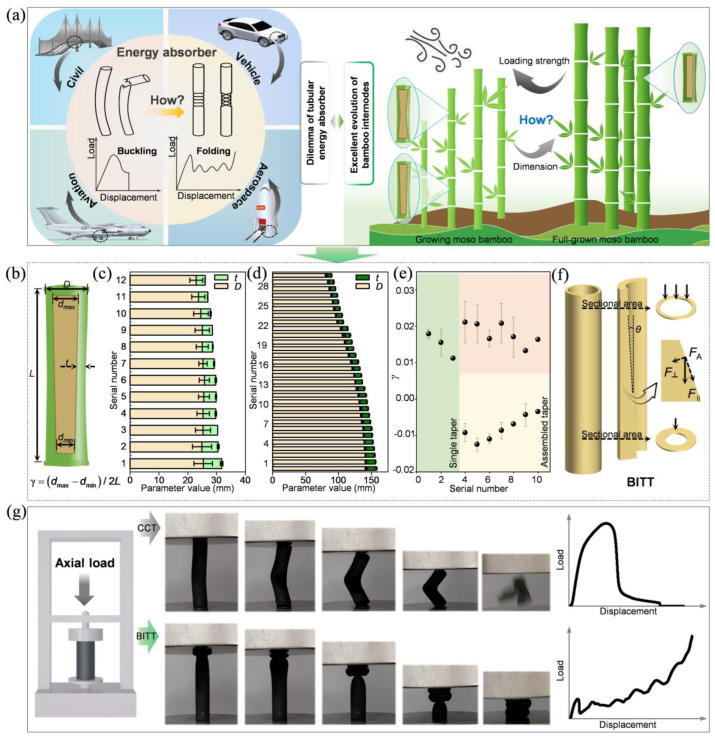
The design inspiration and achieved experimental results of BITT. (**a**) The excellent evolution of bamboo inspires the design of energy absorber. (**b**) The key structural parameters of growing moso bamboo internodes. (**c**) The external diameter (*D*) and average wall thickness (*t*) of growing moso bamboo internodes. (**d**) The *D* and *t* of full-grown moso bamboo internodes. (**e**) The taper (*γ*) of growing moso bamboo internodes. (**f**) Schematic diagram of force decomposition. (**g**) Typical deformation mode and load–displacement curves of BITT and CCT.

Based on the above research background, this study aims to address the technical challenges that tubular structures with large length–diameter ratios are prone to buckling and result in unstable deformation under axial impact, leading to reduced energy absorption performance. Meanwhile, through an in-depth investigation into the energy absorption mechanisms of growing moso bamboo internodes, a bioinspired structural design strategy focused on practical engineering needs is proposed (Figure 1a). The average height of growing bamboo and mature moso bamboo can reach approximately 2 m and 10 m, respectively. Additionally, their average wall thicknesses can reach up to 25 mm and 145 mm, respectively. Under identical environmental conditions, the axial loading strength of growing bamboo is about 12 times greater than that of mature bamboo. Detailed observations and measurements reveal a significant tapered inner wall in the internodes of growing bamboo (Figure 1b). The main geometric characteristics indicate that the wall thickness of both growing and mature bamboo internodes decreases along the natural growth direction (i.e., from the root to the top). However, two completely distinct strategies have evolved to achieve this (Figure 1c,d). Mature moso bamboo reduces wall thickness through the simultaneous decrease in both external and internal diameters of its internodes. In contrast, measurements show that the external diameter of growing bamboo internodes remains essentially constant. Consequently, the gradient reduction in wall thickness in growing bamboo is achieved solely through variations in the internal diameter. By performing multi-point measurements of the internal diameter of growing moso bamboo internodes, the taper of the inner walls was calculated for all internodes (Figure 1e). It was found that the internodes of growing bamboo generally exhibit a tapered inner wall characteristic, with the taper value remaining stable within a specific range (from −0.02 to 0.02). Inspired by this observation, a novel bionic inner-tapered tube (BITT) was designed (Figure 1f). The BITT achieves enhanced material utilization efficiency and energy absorption performance through a progressively improved folding deformation mechanism (Figure 1g). Unlike the conventional outer-tapered tube (COTT) [28], the BITT demonstrates superior axial mechanical properties, making it more suitable as a structural unit in energy-absorbing array sandwich structures. Compared to the conventional circular tube (CCT), the BITT achieves increases in specific energy absorption (SEA) and effective stroke ratio (ESR) of up to 389.39% and 38.10%, respectively. Furthermore, when implemented in square and hexagonal arrays, the introduction of BITT results in SEA increases of 110.77% and 388.92%, respectively, compared to the conventional circular tube arrays.

## 2. Materials and Methods

### 2.1. Characterization of Internode Morphology

Moso bamboos (*Phyllostachys edulis*) in different growth periods (the growing stage and full-grown stage) were chosen as research objects. The bamboo specimens were collected from Shu Yang County, Suqian Municipality, Jiangsu Province, China. The specimen collection area is a warm temperate monsoon climate, with an average annual temperature of 14.1 °C, an average annual relative humidity of 75%, and an average annual sunshine duration of 2046.0 h. The bamboo specimens were cut according to the position of the nodes, and the key structural dimensions were carefully measured with an electronic digital indicator (AIRAJ ARZ1331). Considering the credibility of the data, six bamboo specimens were measured. The measurements involved in this work were repeated three times and averaged.

### 2.2. Design Parameters of BITT

According to the geometric parameters of the growing bamboo internode, a series of BITTs with different taper were constructed. Only three dimensionless parameters (length-diameter ratio (*α*), diameter-thickness ratio (*β*), and taper of inner-wall (*γ*)) and the length (*L*) are needed to determine a BITT. These dimensionless parameters can perfectly imitate the structural characteristics of bamboo internode without the interference of actual size. The CCT of equal volume was built to compare their mechanical behavior. The detailed design process of the specific parameters of BITT and CCT is as follows:

According to the set *L* and *α*, the *D* of BITT can be calculated, and combining with the set *β*, the *t* at the longitudinal middle position of BITT can also be calculated as follows:

(1) $$D=\frac{L}{\alpha }$$

(2) $$t=\frac{D}{\beta }$$

Then, the internal diameter (*d*_1/2_) at the longitudinal middle position of BITT can be calculated as

(3) $$d_{1/2}=D-t$$

According to the set *γ*, the following simultaneous equations regarding the maximum internal diameter (*d*_max_) and minimum internal diameter (*d*_min_) can be listed to solve them:

(4) $$\gamma =\frac{d_{\max}-d_{\min}}{L}$$

(5) $$d_{1/2}=\frac{d_{\max}+d_{\min}}{2}$$

As the control group, the *L* and *D* of CCT are the same as that of BITT. The internal diameter (*d*) can be solved according to the criterion of volume equality; the volume of BITT (*V*_BITT_) and CCT (*V*_CCT_) can be calculated, and according to *V*_BITT_ = *V*_CCT_, the *d* can be obtained as follows:

(6) $$V_{\mathrm{BITT}}=\frac{1}{4}\pi D^{2}L-\frac{1}{12}\pi L\left({d^{2}}_{\max}+{d^{2}}_{\min}+d_{\max}d_{\min}\right)$$

(7) $$V_{\mathrm{CCT}}=\frac{1}{4}\pi L\left(D^{2}-d^{2}\right)$$

(8) $$d=\sqrt{\frac{1}{3}\left({d^{2}}_{\max}+{d^{2}}_{\min}+d_{\max}d_{\min}\right)}$$

The parameter information of all BITTs and related structures is listed (Table 1).

### 2.3. Performance Indicators

For the energy absorption behavior of tubular structure under axial load, its energy absorption efficiency (*f*) can be defined as follow, where *s* is the compression displacement; *F*(*s*) is the compression load, and *F*_max_ is the maximum compression load within [0, *s*] except the initial peak load. The compression displacement corresponding to the maximum *f* will be recorded as the effective compression stroke (*S*_ef_). The effective stroke ratio (ESR) is obtained by dividing the *S*_ef_ by *L*:

(9) $$f=\frac{\int_{0}^{s}F(s)ds}{F_{\max}}$$

(10) $$\mathrm{ESR}=\frac{S_{\mathrm{ef}}}{L}$$

where *L* is the original length of tubes. ESR is a dimensionless evaluation index that reflects the effective utilization of materials in energy-absorbing structures. The specific energy absorption (SEA) per unit weight is obtained by dividing the total absorbed energy within the *S*_ef_ by the total mass (*m*) of the structure:

(11) $$\mathrm{SEA}=\frac{\int_{0}^{S_{\mathrm{ef}}}F(s)ds}{m}$$

### 2.4. Fabrications and Experiment Setup

The sample pieces were fabricated by additive manufacturing, and the 3D printer (Union Tech Lite800, which sourced from Union Tech, located in Shanghai, China.) was chosen for this purpose. A total of three substrate fabrication materials (AlSi10Mg, nylon and resin) were used to screen for a more suitable substrate material. The three selected materials are commonly used for the fabrication of impact-resistant structures, as they exhibit excellent strength and toughness while maintaining a high elongation rate, providing a foundation for large deformation in bioinspired structures. Moreover, these materials are currently among the most mature consumables in the field of 3D printing, ensuring high manufacturing precision. Quasi-static crushing experiments were implemented using the universal material testing machine (SUNS UTM4304X, which was manufactured by SUNS, based in Shenzhen, China.). The bottom of the specimen was put on the support platform, and the quasi-static crushing load was applied with the pressure head that moves down at a constant velocity of 5 mm/min. When the specimen completely failed, the crushing loading was terminated. The deformation process was recorded using a digital camera, and its own system was used to collect the crushing load and displacement data. Five specimens were prepared and tested repeatedly for each type of structure, and the error bar was applied to ensure the accuracy of the results.

### 2.5. Finite Element Model (FEM)

**Figure 2:**
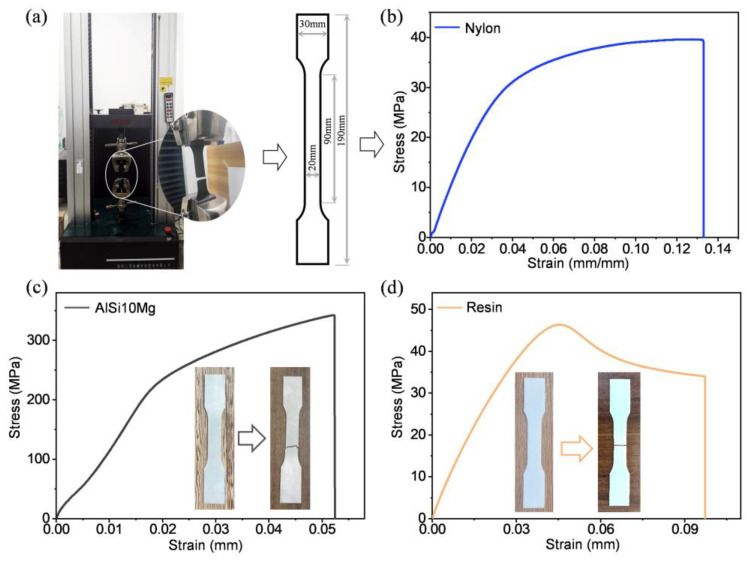
Quasi-static tensile test and typical engineering stress–strain curves of the nylon, AlSi10Mg, and resin. (**a**) Test machine and the specimen size. (**b**) The typical stress–strain curve of nylon. (**c**) The typical stress–strain curve of AlSi10Mg. (**d**) The typical stress–strain curve of resin.

**Figure 3:**
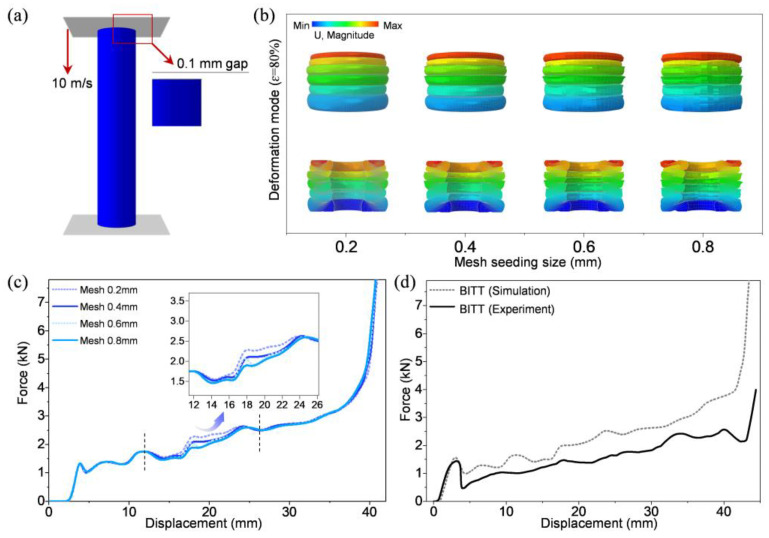
The validation of the finite element model. (**a**) Finite element model. (**b**) The deformation mode of different mesh seeding sizes. (**c**) The force-displacement curves of different mesh seeding sizes. (**d**) The force-displacement curves of BITT obtained via simulation and experiment.

The crashworthiness of BITT under out-of-plane compression is investigated computationally using ABAQUS/Explicit 6.14. The isotropic elastic-plastic model is used for the finite element numerical model based on the true stress–strain curves of the standard tensile test. The properties of three substrate fabrication materials used in this work were obtained via tensile tests of samples (Figure 2). Five tensile specimens were tested using SUNS UTM4304X, and the velocity of the tensile test was 5 mm/min. The corresponding material parameters are shown in Table 2. The FEM includes the bottom rigid plate and the impact rigid plate (Figure 3a). In the initial step, the sample was placed on the rigid plate at the bottom. The impact rigid plate was above the sample with a void of 0.1 mm to simulate the actual impact working condition. An inertial mass of 1 kg was given to the impact rigid plate. The bottom rigid plate was constrained in all directions. The impact rigid plate only retained movement in the axial direction. An initial velocity of 1 m/s was given to the impact rigid plate. The sample was meshed using a C3D8R with an 8-node linear brick, with reduced integration and hourglass control. In a finite element analysis, hourglass control is a numerical technique used to address hourglass modes that may arise when using first-order elements (e.g., quadrilateral or hexahedral elements). Hourglass modes are non-physical zero-stiffness deformation patterns, typically occurring when elements are insufficiently constrained or when the load distribution is unreasonable. These modes can lead to unstable or distorted computational results. Hourglass control suppresses these non-physical modes by introducing artificial stiffness or viscous damping terms within the elements, thereby enhancing the stability and accuracy of the computations. The bottom rigid plate and the impact rigid plate were meshed using the R3D4 with a 4-node 3 D bilinear rigid quadrilateral. The general contact based on a penalty function algorithm was adopted [29,30], and the friction coefficient was set to 0.25 [31,32].

## 3. Results and Discussion

### 3.1. Validation of the FEM

To improve the accuracy of FEM, a sensitivity analysis of the element size was carried out (Figure 3b,c). Several mesh seeding sizes (0.8 mm, 0.6 mm, 0.4 mm, and 0.2 mm) were considered. When the mesh seeding size was too large, some of the meshes were distorted, indicating the inaccuracy of the result. The force-displacement curves were most similar when the mesh seeding sizes were 0.2 mm and 0.4 mm. Therefore, the mesh seeding size of 0.4 mm was selected by balancing the calculated accuracy and calculated cost. The FEM was validated by the experimental results of BITT. The simulation result of the force-displacement curve agreed well with the experimental results (Figure 3d). The force-displacement curves obtained via simulation and experiment demonstrated high consistency in the form of curve and a small numerical difference in the force. This numerical difference may be caused by the manufacturing error of the specimens, the ideal simplification method for FEM. The deformation mode of numerical results was also in good agreement with the experimental results. In general, the simulation results agreed well with the experimental results in deformation mode and in the form of force-displacement. It can be legitimately used to analyze the axial mechanical behavior of BITT. The evaluation indexes of axial mechanical properties in this work were all obtained via experiments.

### 3.2. Optimization of Substrate Materials

**Figure 4:**
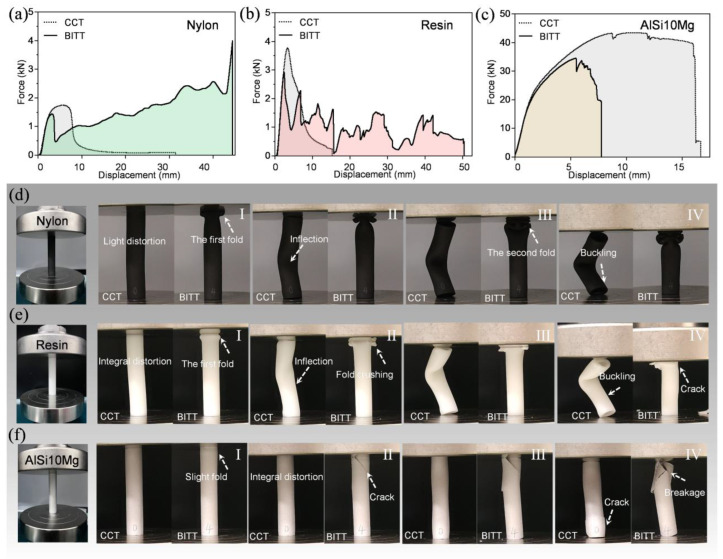
The force-displacement curves and deformation process comparisons of CCT and BITT based on different substrate materials. (**a**–**c**) The force-displacement curves of CCT and BITT fabricated using nylon, resin, and AlSi10Mg. (**d**–**f**) The deformation processes of CCT and BITT fabricated using nylon, resin, and AlSi10Mg.

As the materials often used for energy absorbers, AlSi10Mg, nylon, and resin were selected to screen for a more suitable substrate materials for BITT. The force-displacement curves can preliminarily reflect the energy absorption of BITT compared with CCT with different substrate materials (Figure 4a–c). The initial peak force reflects the load-bearing stability of the structure, while the area under the force-displacement curve represents the absorbed energy. For all the three substrate materials, the force-displacement curve of CCT has only one peak, and its *F*_p_ is higher than that of BITT. Specifically, for nylon, the force-displacement curve of BITT shows multiple peaks, and the peak load is incremental as the displacement increase. The typical deformation processes of CCT and BITT were captured. CCT exhibited a light distortion at first, then inflected in the middle, and finally evolved into buckling. In contrast, the BITT completed the first and second folding deformation, respectively, under the corresponding load stroke (Figure 4d). For resin, the force-displacement curve of BITT shows multiple peaks, and the peak load is diminishing with the increase in displacement. Comparing the deformation mode, CCT exhibited a light distortion at first, then inflected in the middle, and finally evolved into buckling. Correspondingly, the BITT completed the first folding deformation, and then, the fold was crushed, accompanied by cracking (Figure 4e). For AlSi10Mg, the force-displacement curve of BITT is unimodal like CCT, and it has a lower peak load. Comparing the deformation mode, the CCT exhibited a light distortion at first, then cracked at the bottom, which induced failure. The BITT exhibited a light folding at first, then cracked at the top, and the breakage induced the last failure (Figure 4f).

The mechanical characteristics of the curves reflect the energy absorption and deformation modes of each material during the deformation process. The peak force in the curves indicates the strength of the material during the initial deformation phase. The variations in curve shapes for different materials represent their distinct deformation modes. For example, some materials may exhibit pronounced buckling, while others demonstrate progressive folding or crushing behaviors. The force-displacement curves also illustrate the combined effects of material properties and structural design on the energy absorption performance. Based on the above analysis, it can be concluded that BITTs fabricated with three different materials can all improve stability by reducing the *F*_p_ while increasing their energy absorption through enhanced plastic deformation. When the substrate material is nylon or resin, the displacement at failure of BITT increases significantly compared with CCT, leading to an improvement in energy absorption. The energy absorption behavior of resin-based BITT is mainly dominated by structural fragmentation. The BITT made of nylon achieves stable and progressively enhanced folding deformation, which absorbs energy through plastic deformation. Therefore, nylon is selected as the optimized material for maximizing the structural functionality of BITT.

### 3.3. Deformation Mode of BITT

**Figure 5:**
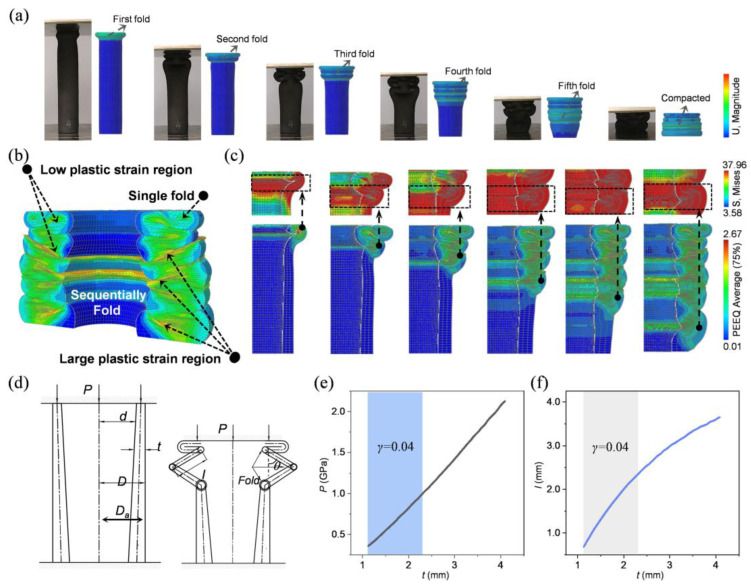
The progressively enhanced fold deformation mode of BITT. (**a**) The typical deformation mode of BITT obtained via experiment and simulation. (**b**,**c**) Typical strain and stress distribution of BITT. The black boxes correspond to the region of stress concentration. (**d**) The progressive enhanced fold deformation schematic drawing. (**e**) The curve of *P*-*t* obtained via theoretical calculation. (**f**) The curve of *l*-*t* obtained via theoretical calculation.

The mechanical performance of a structure is largely governed by its deformation behavior. Based on the experimental results, it can be concluded that the superior performance of the BITT is primarily attributed to the optimization of its deformation mode. Specifically, the BITT demonstrates a progressively enhanced folding deformation mechanism, as evidenced by both simulation and experimental observations. (Figure 5a). This deformation mainly has two characteristics. The first one is the progressively fold deformation. Under axial crushing load, the BITT undergoes sequential deformation from the top to the bottom, forming multiple folds in a progressive manner. This characteristic is clearly reflected in its deformation contours. During the folding process, the regions outside the folding zones exhibit remarkable structural stability. At a strain level of 80%, the deformation mode and the strain distribution of the BITT were thoroughly analyzed (Figure 5b). The bottom folds exhibit a concentration of high plastic strain, whereas the top folds experience relatively lower levels of plastic strain. The second one is the enhancement of fold. During the compaction stage, the gradient wall thickness of the BITT imparts greater structural strength at the bottom compared to the top, causing plastic deformation to initiate preferentially at the top. The volume of each fold progressively increases from top to bottom, indicating an enhancement in the folding deformation. This progressive and amplifying deformation behavior is further validated by the development of stress concentration regions (Figure 5c). Notably, with the formation of each fold, the corresponding region becomes the primary stress concentration zone. The shifting location of these stress concentration regions illustrates the stepwise transfer of forces from the top to the bottom of the BITT, enabling the effective dissipation of axial stress and the controlled attenuation of kinetic energy in a gradual and systematic manner.

An approximate analysis of the collapse of thin cylindrical shells under axial compression load proposed by Alexander can be used to understand the deformation mode of BITT [33]. Combined with Alexander’s theory and mathematical analysis, the progressively enhanced deformation mode of BITT can be verified theoretically. This can be calculated by defining half of each fold deformation as the length of half-fold step (*l*) and the mean crushing load (*P*) to form a complete fold deformation (Figure 5d). The whole derivation process is as follows:

(12) $$dW_{1}=4Md\theta \pi (D_{a}+l\sin\theta )$$

where *M* is the crushing moment of the plastic hinge in unit circumferential length, which can be approximated as a narrow-beam element. *M* can be calculated using *Yt*^2^/4, where *Y* is the yield stress of substrate material under unidirectional tensile or compressive load, and *t* is the wall thickness. *D*_a_ is the middle diameter of the structure. *θ* is the angle between the folded central line and the vertical direction. The beam is assumed to be abnormally wide. When the overall deformation is generated, it can be regarded as a deformation under the basic plane strain condition. If it follows the von Mises yield criterion of materials, and the crushing moment can be calculated as

(13) $$M=\frac{2}{\sqrt{3}}(\frac{Yt^{2}}{4})$$

Hence, Formula (12) can be rewritten as follows:

(14) $$dW_{1}=\frac{2\pi }{\sqrt{3}}Yt^{2}d\theta (D_{a}+l\sin\theta )$$

When the structure deforms *dθ*, its average strain and work generated are calculated as follows:

(15) $$\frac{\pi \left[D_{a}+l\sin(\theta +d\theta )\right]-\pi \left[D_{a}+l\sin\theta \right]}{¦\pi (D_{a}+l\sin\theta )}=\frac{ld\cos\theta }{D_{a}+l\sin\theta }$$

(16) $$dW_{2}=\frac{Yld\theta \cos\theta }{D_{a}+l\sin\theta }\pi (D_{a}+l\sin\theta )(2lt)=2\pi Yl^{2}td\theta \cos\theta$$

By integrating the work performed by *θ* in the transformation range of 0°~90°, the work of a complete plastic hinge formation process can be obtained as follows:

(17) $$W=\frac{1}{n}\int \left(dW_{1}+dW_{2}\right)=\int_{0}^{\frac{\pi }{2}}\left[\frac{2\pi }{\sqrt{3}}Yt^{2}(D_{a}+l\sin\theta )+2\pi Y^{2}t\cos\theta \right]d\theta$$

Assuming a *P*, the work performed in this process divided by the total displacement must be equivalent to

(18) $$P=\frac{W}{2l}=Y\left[\frac{\pi t^{2}}{\sqrt{3}}\left(\frac{\pi D_{a}}{2l}+1\right)+\pi lt\right]$$

There are two unknown values, *P* and *l*, in the above formula, so it hypothesizes that the *P* is minimum, and the half step *l* of the plastic hinge can be calculated as follows:

(19) $$l=\sqrt{\left(\frac{\pi }{2\sqrt{3}}\right)}D_{a}t\approx 0.953\sqrt{D_{a}t}$$

When the *P* is not minimum, the *l* can be expressed as

(20) $$l=k\sqrt{D_{a}t}$$

Here, *k* can only be positive, and plugging this expression into (18), it can be rewritten as follows:

(21) $$P=Y\left[\left(\frac{\pi^{2}}{2\sqrt{3}k}+\pi k\right)\sqrt[{2}]{t^{3}}\sqrt{D_{a}}+\frac{\pi t^{2}}{\sqrt{3}}\right]$$

The deformation mode of the other part is exactly the opposite of the deformation mode derived above, and the structure wall faces inward contraction. If the above derivation process is repeated, the following results are obtained:

(22) $$P=Y\left[\left(\frac{\pi^{2}}{2\sqrt{3}k}+\pi k\right)\sqrt[{2}]{t^{3}}\sqrt{D_{a}}-\frac{\pi t^{2}}{\sqrt{3}}\right]$$

Considering that the actual deformation pattern should be between the inward contraction and outward expansion folds, it is worthwhile to average Formulas (21) and (22) above as follows:

(23) $$P=Y\left[\left(\frac{\pi^{2}}{2\sqrt{3}k}+\pi k\right)\sqrt[{2}]{t^{3}}\sqrt{D_{a}}\right]$$

When the *P* is minimum, *k* can be 0.953, so it can be rewritten as follows:

(24) $$P=5.99Y(\sqrt[{2}]{t^{3}D_{a}})$$

For CCT, the wall thickness is uniform, so the folded fold steps formed are roughly equal. The wall thickness of BITT is characterized by a gradient change in the inner wall taper, which undoubtedly has an important impact on its plastic deformation mode. According to Equation (19), the *l* is simultaneously affected by *D*_a_ and *t*. In BITT, the *D*_a_ can be represented as a function of *t*:

(25) $$D_{a}=D-\frac{t}{2}$$

Hence, the solution of *l* for BITT can be obtained as follows:

(26) $$l=k\sqrt{Dt-\frac{t^{2}}{2}}$$

The minimum *P* solution for BITT can be written as

(27) $$P=5.99Yt\sqrt{Dt-\frac{t^{2}}{2}}$$

According to Formula (12), the *D* of BITT is fixed, so it can be deduced that the *l* and the *P* are determined by the *t*. As *t* increases, the changes in *l* and *P* of BITT are plotted (Figure 5e,f). The *P* increases linearly, while the *l* increases in a parabola. The increase in *P* and *l* verified the previous analysis on the progressively enhanced fold deformation mode. The intine taper of BITT leads to a continuous increase in wall thickness from the top to the bottom, which improves the mean axial load required to form a fold and the folding volume. The main features of the progressively enhanced fold deformation mode were explicated as mentioned above. It must be noted that this theoretical derivation is only a theoretical support for the formation principle of the BITT’s progressively enhanced fold deformation mode. Since the wall thickness of the tapered internal wall changes continuously, the folding deformation needs to divide the entire structure into several parts along its axial length. Thus, the theoretical calculation results roughly predict the *P* and *l*.

Compared with Euler’s buckling of CCT, the progressively enhanced fold deformation mode of BITT reinforces the structure and promotes energy absorption. Due to the progressive fold mode, the structure can always maintain excellent stability. The P that needed to form a fold increases as the displacement loading increases, which strengthens the resistance to damage. The displacement at failure of BITT is extended to about twice that of CCT, which allows for greater plastic deformation to absorb more energy. The unique deformation mode is dominated by the inner wall taper, and the taper optimizes the deformation mode of BITT by adjusting and controlling the axial load transfer path and load-bearing area. To be more specific, the gradient wall slope resulted from the internal wall taper decomposes the load force and makes part of the axial force transfer along the radial direction, which facilitates the structure plastic deformation in the non-axial load direction and promotes energy consumption. Meanwhile, the taper of internal wall of the tube induces a continuous increase in wall thickness, which leads to the load area increase in the axial load transfer path, so the fold deformation volume also increases as axial load loading increases. The progressive transfer path enables BITT to dissipate more intense load via large deformation and force decomposition at the beginning of loading, which enhances the stability and strength of the overall structure by the constant rebuilding of structure.

### 3.4. Taper and Length-Diameter Ratio Effects

**Figure 6:**
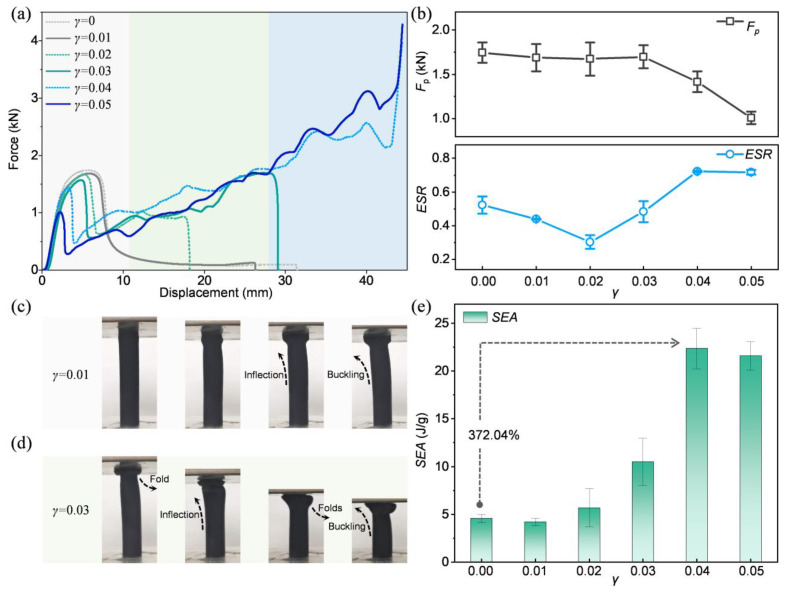
The influence of *γ* on the energy absorption properties of BITT. (**a**) The force-displacement curves of BITT with different *γ*. (**b**) The *F*_p_ and ESR of BITT with different *γ*. (**c**) The deformation mode of BITT with a *γ* of 0.01. (**d**) The deformation mode of BITT with a *γ* of 0.03. (**e**) The SEA of BITT with different *γ*.

As a defining feature of BITT, the taper (*γ*) plays a pivotal role in its energy absorption and stability. To understand its impact, different levels of *γ* (0~0.05) were proposed and investigated. Force-displacement curves were obtained, which reveal three distinct deformation modes for different levels of *γ* (Figure 6a). When *γ* is 0.01, the force-displacement curve shows a single peak, indicating a buckling failure mode (Figure 6c). When *γ* is 0.02 or 0.03, the curve exhibits a few peaks before abruptly descending at a specific displacement, with the force dropping to zero. This behavior signifies a toppling failure, preceded by partial folding and buckling (Figure 6d). In contrast, for *γ* at 0.04 or 0.05, the force-displacement curve displays multiple peaks and a sharp increase during loading, characteristic of a progressively enhanced folding deformation mode. The change of *F*_p_ and ESR with different *γ* was obtained (Figure 6b). As the *γ* increases, the *F*_p_ decreases and has a more significant reduction at large *γ*. However, as the *γ* increases, the ESR first decreases and then increases. When the *γ* is 0.05, the BITT shows the minimum *F*_p_ and the maximum ESR. The SEA of the BITTs was calculated (Figure 6e). As the *γ* increases, the SEA increases and has a more significant augment at large *γ*. In summary, when the *γ* is larger than 0.01, the deformation mode of BITT is transformed from buckling to partially folding, and its various performance indexes are slightly improved. When the *γ* is larger than 0.03, the deformation mode of BITT results in progressively enhanced folds, and its performance indicators are significantly enhanced. The maximum SEA is 389.39% higher than that of the CCT.

**Figure 7:**
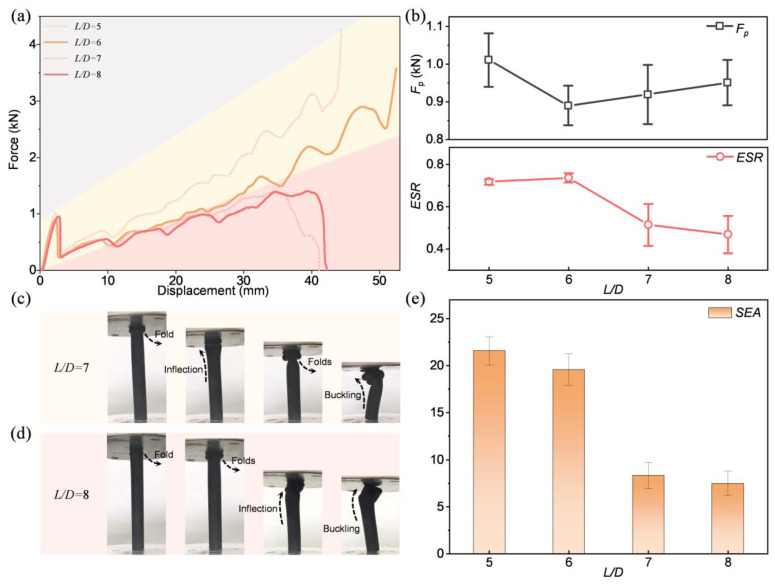
The influence of *L*/*D* on energy absorption properties of BITT. (**a**) The force-displacement curves of BITT with different *L*/*D*. (**b**) The *F*_p_ and ESR of BITT with different *L*/*D*. (**c**) The deformation mode of BITT with a *L*/*D* of 7. (**d**) The deformation mode of BITT with a *L*/*D* of 8. (**e**) The SEA of BITT with different *L*/*D*.

For tubular structures, the length–diameter ratio (*L*/*D*) is a main factor limiting their axial energy absorption and structural stability. According to the previous conclusion, when the *γ* is 0.05, BITT has the smallest *F*_p_, the largest ESR, and near-maximum SEA, which are considered the optimal taper. Therefore, BITT with a *γ* of 0.05 was taken as the basis control. Its *L*/*D* was increased to six, seven, and eight, respectively, to study the effects of *L*/*D* on the axial energy absorption. The force-displacement curves of BITTs with different *L*/*D* were obtained (Figure 7a). It can be divided into two types corresponded to two typical deformation modes. When the *L*/*D* is seven or eight, there are a few peaks in its force-displacement curve. Until the displacement is loaded to a certain value, the curve suddenly descends, and the force value decreases to zero. It means the structure topples, and it partially folded and buckled before toppling (Figure 7d,e). When the *L*/*D* is five or six, the progressively enhanced fold deformation mode is developed. The change of *F*_p_ and ESR with different *L*/*D* was obtained (Figure 7b). As the *L*/*D* increases, the *F*_p_ first decreases and then increases, with an unobvious value change. Meanwhile, the ESR first increases and then decreases, with a significant value change. When the *L*/*D* is six, BITT shows the minimum *F*_p_ and the maximum ESR. The SEA of BITT was obtained (Figure 7e). As the *L*/*D* increases, the SEA decreases with a significant diminution at large *L*/*D*. However, when the *L*/*D* is six, the SEA decreases by only 9.23%. It can be inferred that BITT achieves significant improvement of axial mechanical properties within a certain range of *L*/D (e.g., 5~6). When the *L*/*D* exceeds a certain value, the performance of the improvement effect will be weakened (e.g., 7~8). Furthermore, when the required *L*/*D* of BITT is relatively large, its axial mechanical properties can be improved by appropriately increasing the *γ*.

### 3.5. BITT-Based Energy-Absorbing Sandwich

**Figure 8:**
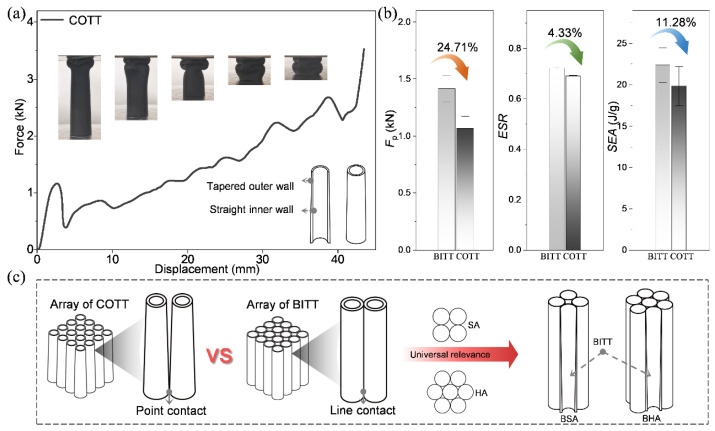
BITT as a structural unit to compose energy-absorbing sandwich. (**a**) The structural representation and force-displacement curve of COTT. (**b**) The comparison of *F*_p_, ESR, and SEA between BITT and COTT. (**c**) The structural representation of the arrays that take BITT as the basic structural unit.

Energy-absorbing sandwich can be obtained by arraying the BITTs to expand its potential engineering applications. To investigate the excellence of BITT as a structural unit of energy-absorbing sandwich, the COTT was established as a contrast. The axial mechanical properties of COTT were tested. The results show that COTT developed similar force-displacement curves and deformation patterns with BITT (Figure 8a). The *F*_p_ of COTT was reduced by 24.71% compared to BITT, which represents the improvement of structural-bearing stability. However, its ESR and SEA decreased by 4.33% and 11.28%, respectively, when compared to that of BITT (Figure 8b). The comparison of performance indicators shows the mechanical advantages of COTT and BITT. The COTT has better stability, while the BITT has a higher energy absorption efficiency. Furthermore, as a basic structural unit to form an energy-absorbing sandwich, the structural excellence of BITT is reflected. The contact forms between the structural units of COTT and BITT arrays are point contact and line contact, respectively (Figure 8c). The line contact allows for more interactions between structural units, which can improve the mechanical properties of the array and have universal relevance. Furthermore, bionic square array (BSA) and bionic hexagon array (BHA) were obtained by arraying BITT in the form of a square and a regular hexagon, respectively. Their axial mechanical properties were investigated by taking the square array (SA) and hexagonal array (HA) composed of conventional circular tubes as the control.

**Figure 9:**
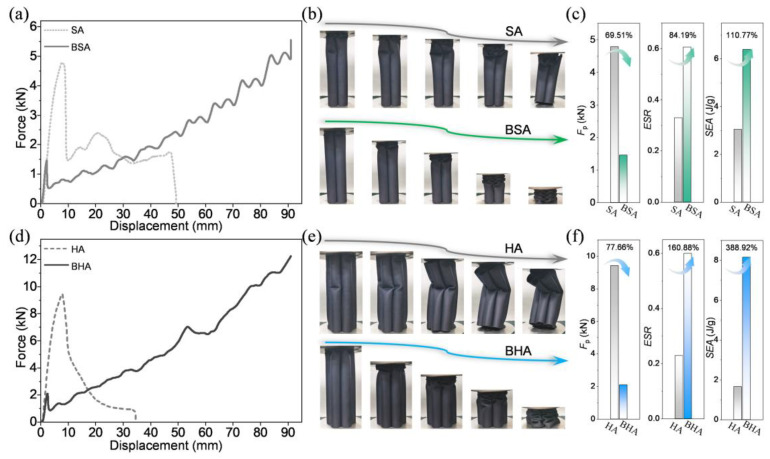
The excellent axial mechanical properties of BSA and BHA. (**a**) The force-displacement curves of SA and BSA. (**b**) The deformation modes of SA and BSA. (**c**) The *F*_p_, ESR, and SEA of SA and BSA. (**d**) The force-displacement curves of HA and BHA. (**e**) The deformation modes of HA and BHA. (**f**) The *F*_p_, ESR, and SEA of HA and BHA.

The force-displacement curve of SA only has a few peaks, and it ends with a sudden drop, while the force-displacement curve of BSA has multiple peaks with increasing force values (Figure 9a). The difference in the force-displacement curves depends on the deformation modes (Figure 9b). The SA is buckled after several initial irregular folds, while the deformation mode of BSA results in progressively enhanced fold. Comparing the performance indicators of the two arrays, all the performance indicators of BSA are significantly optimized. The *F*_p_ decreased by 69.51%, while the ESR and SEA improved by 84.19% and 110.77%, respectively (Figure 9c). It can be inferred that the axial mechanical properties of BSA have been comprehensively and significantly improved. It is surprising that a more significant improvement was found in the BHA. Due to the increase in structural units, the force-displacement curve of HA has only one peak, and its deformation mode is buckling without any folding. By contrast, BHA can still develop progressively enhanced fold deformation mode, and the performance indicators are more significantly optimized (Figure 9d,e). Compared with HA, the *F*_p_ decreased by 77.66%, while the ESR and SEA improved by 160.88% and 388.92%, respectively (Figure 9f). Thus, based on the structural characteristics and the progressively enhanced fold deformation mode of BITT, it can be used to form sandwiches to improve the bearing stability and energy absorption efficiency. The excellent mechanical properties of sandwich based on the BITT prove its usability in a broad engineering field.

**Figure 10:**
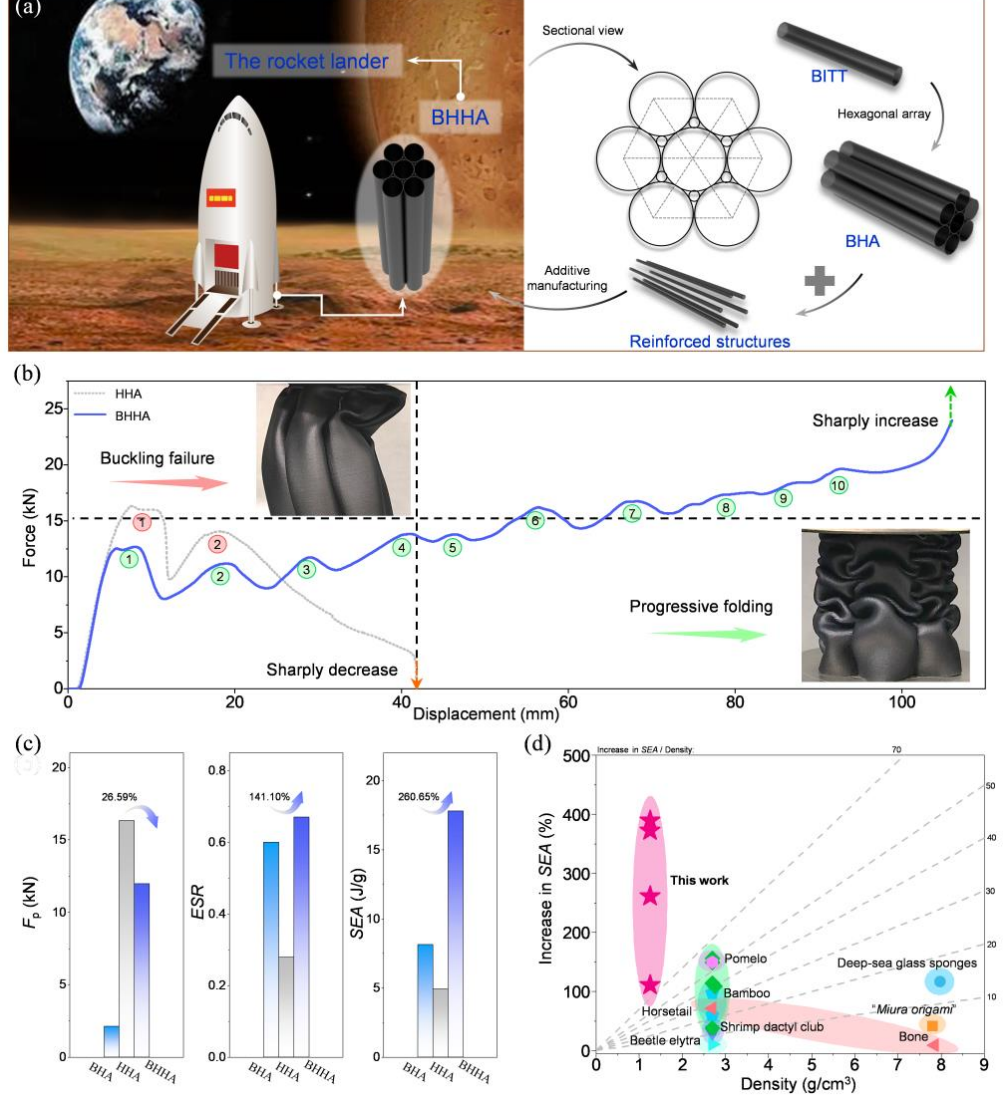
A possible application of BITT as a bionic landing leg in a rocket lander. (**a**) Structural composition diagram of BHHA. (**b**) The force-displacement curve comparison of BHHA and HHA. The numbers in the figure represent the number of peaks in the force curve. (**c**) The comparison of *F*_p_, ESR, and SEA between BHA, HHA, and BHHA. (**d**) Ashby chart summarizing the increase in SEA in this work and other bionic structures in the recent years. The different color blocks represent bionic structures inspired by different biological sources. For details, please refer to the following references: Bamboo [24,26,34], Shrimp dactyl club [29], Beetle elytra [30,35], “*Miura origami*” [31], Deep-sea glass sponges [32], Beetle elytra [30,35], Pomelo [36], Horsetail [37,38], Bone [39,40].

Based on the excellent energy absorption efficiency and the structural load stability of BITT, a bionic hierarchical hexagonal array (BHHA) was designed as the landing leg of rocket landers, which is constituted by BHA and reinforced structures (Figure 10a). For the conventional hierarchical hexagonal array (HHA), Euler’s buckling is the main failure mode due to its large slenderness ratio (Figure 10b). The BHHA forms a perfect progressive folding deformation mode and possesses a high structural strength until completely compacted. The load–displacement curves show that HHA forms two force peaks then the force sharply decreases to zero. However, the BHHA forms 10 force peaks then the force sharply increases, which not only delays crushing failure but also improves structure strength by the constant rebuilding of the structure. The security of BHHA is significantly enhanced. Compared with HHA, the *F*_p_ of BHHA decreased by 26.59%, while the ESR and SEA increased by 141.10% and 260.65% (Figure 10c). In addition, comparing with BHA, the reinforced structures of BHHA can improve the ESR and SEA, but the increase of *F*_p_ indicates a weakened stability. Therefore, it is necessary to selectively add reinforced structures according to actual engineering requirements. The increase in SEA of this work and other bionic structures was compared to evaluate the performance improvement efficiency. It is intuitively found that the BITT and BITT-based energy absorption arrays achieved more significant improvements in SEA (Figure 10d). The excellent energy absorption performance of BHHA as a rocket landing leg demonstrates the great potential of BITT in various similar working conditions. For example, it can be applied to automobile bumpers to improve crash resistance or to the landing gear of large aircraft to enhance safety and stability factors. At the same time, its structure is simple and achieves dimensionless parameterization, allowing for the preparation of samples at different scales through various methods according to different working conditions. This greatly reduces the difficulty of practical application of BITT.

## 4. Conclusions

In conclusion, the discovery of the tapered intine inside moso bamboo reveals its critical role in providing exceptional axial mechanical properties as a natural slender structure. This taper enables growing moso bamboo to withstand severe impacts comparable to those endured by stout, full-grown moso bamboo. Inspired by this natural feature, a bionic inner-tapered tube (BITT) was rationally designed. The mechanical behavior and energy absorption performance of BITT were evaluated through quasi-static axial compression tests, supported by theoretical calculations and finite element analysis to comprehensively elucidate its axial energy absorption mechanisms. The BITT exhibits a progressively enhanced fold deformation mode, which effectively prevents buckling failure, reduces the initial peak crushing load, and significantly improves energy absorption efficiency by optimizing plastic deformation mode. Nylon was identified as the optimal base material through comparative tests. The effects of taper and length-diameter ratio on the axial energy absorption of BITT were systematically investigated. When the γ is larger than 0.03, the deformation mode of BITT results in typical progressively enhanced fold. Its performance indicators were significantly enhanced, and the maximum SEA is 389.39% higher than that of the CCT. The BITT achieves significant improvement of axial energy absorption within a certain range of *L*/D (e.g., 5~6). When the *L*/*D* exceeds a certain value, the performance improvement effect will be weakened (e.g., 7~8). The BITT-based BSA and BHA showed excellent axial mechanical strength and energy absorption, which can be used to form sandwiches to improve the bearing stability and energy absorption efficiency. Lastly, the BITT-based hierarchical structure was proposed to be applied to the landing leg of a rocket to improve its safety. The BITT and the related derivative structures achieve a significant improvement in axial energy absorption and stability.

The bionic design methodology developed in this study provides an effective approach to improve the axial energy absorption efficiency and stability of slender tubes as energy absorbers. By leveraging macrostructure design, this work aims to overcome the inherent limitations of substrate materials. This strategy is expected to accelerate the adoption of composite materials in critical components of major engineering systems in the future.

## Figures and Tables

**Table 1:** Designation and dimensions of different models used in this work.

Model Identification	*L* (mm)	*D* (mm)	*γ*
BITT	60, 72, 84, 96	12	0.01, 0.02, 0.03, 0.04, 0.05
CCT	60	12	0.00
COTT	60	12	0.05
Structural unit of bionic arrays	150	20	0.013

**Table 2:** Material parameters of three substrate materials.

Material Categories	Young’s Modulus (*E*/MPa)	Yield Stress (σ_s_/MPa)	Ultimate Stress (σ_u_/MPa)	Poisson’s Ratio (*ν*)	Density (*ρ*/g/cm^3^)
Nylon	1016.21	21.40	39.76	0.33	1.30
Resin	1277.32	42.70	48.47	0.33	1.25
AlSi10Mg	12363.35	228.81	360.29	0.33	2.70

## Data Availability

The original contributions presented in this study are included in the article. Further inquiries can be directed to the corresponding author.
